# Quabodepistat in combination with delamanid and bedaquiline in participants with drug-susceptible pulmonary tuberculosis: protocol for a multicenter, phase 2b/c, open-label, randomized, dose-finding trial to evaluate safety and efficacy

**DOI:** 10.1186/s13063-024-07912-5

**Published:** 2024-01-19

**Authors:** Rodney Dawson, Andreas H. Diacon, Simbarashe Takuva, Yongge Liu, Bo Zheng, Vatsala Karwe, Jeffrey Hafkin

**Affiliations:** 1 Division of Pulmonology, Department of Medicine, University of Cape Town and University of Cape Town Lung Institute, Cape Town, South Africa; 2 Department of Medicine, Stellenbosch University, Cape Town, South Africa; 3 TASK Applied Science, Cape Town, South Africa; 4 Otsuka Novel Products GmbH, Munich, Germany; 5 Faculty of Health Sciences, School of Health Systems and Public Health, University of Pretoria, Pretoria, South Africa; 6 Otsuka Pharmaceutical Development & Commercialization, Inc., Rockville, MD USA

**Keywords:** Antituberculosis agent, Bedaquiline, Delamanid, DprE1 inhibitor, Drug-sensitive tuberculosis, Quabodepistat, Tuberculosis

## Abstract

**Background:**

Delamanid and bedaquiline are two of the most recently developed antituberculosis (TB) drugs that have been extensively studied in patients with multidrug-resistant TB. There is currently a need for more potent, less-toxic drugs with novel mechanisms of action that can be used in combination with these newer agents to shorten the duration of treatment as well as prevent the development of drug resistance. Quabodepistat (QBS) is a newly discovered inhibitor of decaprenylphosphoryl-β-D-ribose-2′-oxidase, an essential enzyme for *Mycobacterium tuberculosis* to synthesize key components of its cell wall. The objective of this study is to evaluate the safety, efficacy, and appropriate dosing of a 4-month regimen of QBS in combination with delamanid and bedaquiline in participants with drug-susceptible pulmonary TB in comparison with the 6-month standard treatment (i.e., rifampicin, isoniazid, ethambutol, and pyrazinamide).

**Methods:**

This phase 2b/c, open-label, randomized, parallel group, dose-finding trial will enroll approximately 120 participants (including no more than 15% with human immunodeficiency virus [HIV] coinfection) aged ≥ 18 to ≤ 65 years at screening with newly diagnosed pulmonary drug-sensitive TB from ~8 sites in South Africa. Following a screening period of up to 14 days, eligible participants will be randomized in a ratio of 1:2:2:1 to one of four arms. Randomization will be stratified by HIV status and the presence of bilateral cavitation on a screening chest x-ray. After the end of the treatment period, participants will be followed until 12 months post randomization. The primary efficacy endpoint is the proportion of participants achieving sputum culture conversion in Mycobacteria Growth Indicator Tube by the end of the treatment period. The safety endpoints consist of adverse events, clinical laboratory tests, vital signs, physical examination findings, and electrocardiographic changes.

**Discussion:**

QBS’s potent bactericidal activity and distinct mechanism of action (compared with other TB drugs currently available for human use) may make it an ideal candidate for inclusion in a novel treatment regimen to improve efficacy and potentially prevent resistance to concomitant TB drugs. This trial will assess the effectiveness, safety, and dosing of a new, shorter, QBS-based, combination anti-TB treatment regimen.

**Trial status:**

ClinicalTrials.gov NCT05221502. Registered on February 3, 2022

**Supplementary Information:**

The online version contains supplementary material available at 10.1186/s13063-024-07912-5.

## Introduction

### Background and rationale

Tuberculosis (TB), the communicable disease caused by the acid-fast bacillus *Mycobacterium tuberculosis*, remains a leading cause of mortality by an infectious disease worldwide despite the availability of an array of curative treatments [1, 2]. In 2021, it was estimated that 10.6 million people became ill with TB, and 1.6 million people died from it [1]. The most common manifestation of active infection is pulmonary TB [1].

Standard treatment for drug-susceptible TB is a 6-month regimen of anti-TB agents, including rifampicin, isoniazid, ethambutol, and pyrazinamide (RHEZ), which are prescribed to not only rapidly reduce the replicating bacillary population and eradicate dormant or persisting bacilli but also to prevent the acquisition of drug resistance during therapy [2–4]. However, resistance to available drugs is increasingly common, with 3.6% of new cases and 18% of previously treated cases demonstrating multidrug resistance to at least isoniazid and rifampicin [1]. This has sparked an interest in the development of newer oral agents to treat patients with multidrug-resistant TB, including nitroimidazole delamanid (DLM) and diarylquinoline bedaquiline (BDQ) [5–7]. There is currently a need for more potent, less-toxic drugs with novel mechanisms of action that can be used in combination with these newer agents to shorten the duration of treatment, combat the development of resistance, and be broadly used to transition from multidrug resistant to drug-susceptible TB.

The 3,4-dihydrocarbostyril derivative quabodepistat (QBS; OPC-167832) is a new oral anti-TB agent that has a mechanism of action that is distinct from currently available anti-TB therapies; that is, it inhibits decaprenylphosphoryl-β-D-ribose-2′-oxidase (DprE1), an enzyme essential for *M. tuberculosis* cell-wall biosynthesis [8, 9]. Moreover, it has demonstrated high anti-TB activity both in vitro and in various animal TB models and in an early bactericidal activity trial in patients with drug-susceptible pulmonary TB [8, 10–12].

The proposed treatment regimen of DLM, BDQ, and QBS has supportive data from both nonclinical and clinical studies that have examined these treatments alone and in combination. In both a mouse chronic TB model and hollow fiber system of tuberculosis, the three-drug combination of DLM, BDQ, and QBS demonstrated more rapid microbiologic response compared with the standard treatment, RHEZ [8, 13]. In addition, a 14-day early bactericidal study of the DLM/BDQ/QBS regimen has also been performed in patients with drug-susceptible TB and suggests a comparable antibacterial response to RHEZ and a favorable safety profile [12].

### Objective

The primary objective of this phase 2b/c, open-label, randomized, dose-finding trial is to evaluate the safety and efficacy of a 4-month regimen of QBS in combination with DLM and BDQ in participants with drug-susceptible pulmonary TB in comparison with standard treatment (RHEZ). This trial will help identify the optimal dose of QBS and determine the suitability of the three-drug combination as a backbone to construct new regimens for use in future TB studies. It is expected that the development of new efficient regimens with shorter treatment durations will help simplify TB therapy, enhance compliance, and potentially improve outcomes.

### Trial design

In this multicenter, phase 2b/c, open-label, parallel group, randomized, dose-finding, single-country (South Africa) trial, eligible participants with drug-susceptible TB, after a screening period of up to 14 days, will be randomized on day 1 or pre-dose day 1 in a ratio of 1:2:2:1 to one of the following treatment arms:

1. DBQ10: DLM (300 mg once daily [QD]) + BDQ (400 mg QD × 2 weeks and then 200 mg thrice weekly [TIW]) + QBS (10 mg QD) for 17 weeks
2. DBQ30: DLM (300 mg QD) + BDQ (400 mg QD × 2 weeks and then 200 mg TIW) + QBS (30 mg QD) for 17 weeks
3. DBQ90: DLM (300 mg QD) + BDQ (400 mg QD × 2 weeks and then 200 mg TIW) + QBS (90 mg QD) for 17 weeks
4. RHEZ: RHEZ for 8 weeks followed by 18 weeks of rifampin and isoniazid (for a total of 26 weeks)

**Fig. 1:**
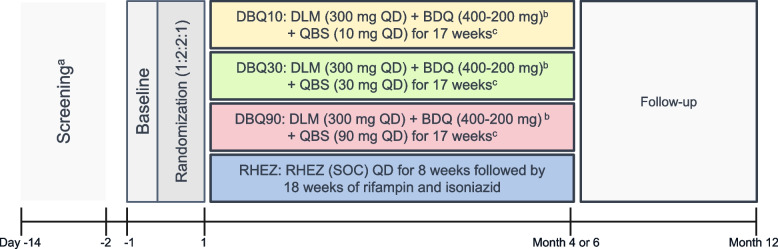
Trial design. ^a^Screening can occur for up to 14 days but should be completed in as few days as possible. ^b^BDQ dose 400 mg QD × 2 weeks and then 200 mg TIW. ^c^Rescue treatment consisting of the standard of care treatment, i.e., RHEZ, will be provided to participants in DBQ10, DBQ30, and DBQ90 treatment arms in the following circumstances: (1) If a participant is sputum culture positive for *M. tuberculosis* at the end of treatment, (2) if a participant has a microbiologic relapse subsequent to the end of treatment, or (3) if, in the opinion of the investigator, a participant is clinically deteriorating, based on any combination of signs, symptoms, or chest radiograph data. Participants in the RHEZ arm will be managed according to local guidelines if not responding to therapy. BDQ, bedaquiline; DBQ, delamanid+bedaquiline+quabodepistat; DLM, delamanid; QBS, quabodepistat; QD, once daily; RHEZ, rifampicin, isoniazid, ethambutol, and pyrazinamide; SOC, standard of care; TIW, thrice weekly

At the end of treatment, participants will be in follow-up until 52-week post-randomization. A trial design schema is shown in Fig. 1.

## Methods: participants, interventions, and outcomes

### Study setting

This multiple-dose trial will be conducted at approximately eight trial sites in South Africa (Supplemental Table 1). Sites were chosen based on their experience in conducting drug-susceptible pulmonary TB trials, enrollment capabilities, infection control, contract timelines, and Institutional Review Board (IRB)/local government unit approval timelines.

### Eligibility criteria

The trial population will consist of participants who are ≥ 18 years to ≤ 65 years at the time of screening and who have newly diagnosed pulmonary drug-susceptible TB. Approximately 120 participants (including no more than 15% with human immunodeficiency virus [HIV] coinfection) who meet all of the inclusion criteria and none of the exclusion criteria listed in Table 1 will be eligible for study participation.

**Table 1:** Inclusion and exclusion criteria

Inclusion criteria	
**•** Able to provide written, informed consent prior to initiation of any trial-related procedures or treatments and able, in the opinion of the investigator, to comply with all the requirements of the trial **•** Male or female participants between 18 and 65 years of age (inclusive) at the screening visit **•** Body weight ≥ 35.0 kg at the screening visit **•** Newly diagnosed rifampin and isoniazid susceptible (on the screening sample) pulmonary tuberculosis (TB) **•** Able to spontaneously produce sputum **•** Females of childbearing potential (FOCBP) must agree to use two different approved methods of birth control or remain abstinent throughout the participation in the trial and for 12 weeks after the last dose of study drug or dose of RHEZ **•** Male participants must agree to use two different approved methods of birth control or remain abstinent throughout the participation in the trial and for 12 weeks after the last dose of study drug or RHEZ	
Exclusion criteria	
**•** Participants are known or suspected of having resistance to rifampin, isoniazid, ethambutol, pyrazinamide, DLM, or BDQ either confirmed by the laboratory or based on epidemiological history, at screening **•** Evidence of clinically significant metabolic (e.g., including ongoing or current hypokalemia [i.e., potassium < 3.5 mEq/dL at screening]), gastrointestinal, neurological, psychiatric, endocrine, or liver (e.g., hepatitis B and C) disease, malignancy, or other abnormalities (other than the indication being studied) **•** History of, or current, clinically relevant cardiovascular disorder such as heart failure, coronary heart disease, uncontrolled hypertension, arrhythmia or symptom strongly suggestive of such a problem (e.g., syncope or palpitations), tachyarrhythmia, or status after myocardial infarction **•** Known bleeding disorders or family history of bleeding disorders **•** Any diseases or conditions in which the use of DLM, BDQ, quabodepistat (QBS), rifampin, isoniazid, pyrazinamide, or ethambutol is contraindicated **•** Any prior treatment for *M. tuberculosis* within the past 2 years **•** Any treatment with a drug active against *M. tuberculosis* (e.g., quinolones) within the 3 months prior to screening **•** Clinical evidence of severe extrapulmonary TB (e.g., miliary TB, abdominal TB, urogenital TB, osteoarthritic TB, TB meningitis) **•** Evidence of pulmonary silicosis, lung fibrosis, or other lung condition considered as severe by the investigator (other than TB). In particular, any underlying condition that could interfere with the assessment of x-ray images, sputum collection, or interpretation of sputum findings, or otherwise compromise the subject’s participation in the trial **•** Any renal impairment characterized by creatinine clearance/estimated glomerular filtration rate (eGFR) of < 60 mL/min/1.73 m^2^, or hepatic impairment characterized by alanine transaminase or aspartate transaminase > 2.0 × upper limit of normal of the clinical laboratory reference range, or bilirubin > 2.0 × upper limit of normal of the clinical laboratory reference range, at screening **•** Screening glucose (non-fasting) ≥ 200 mg/dL or glycosylated hemoglobin (HbA1c) ≥ 6.5% **•** QTcF > 450 msec in male participants (> 470 msec in female participants), atrioventricular block II or III, bi-fascicular block, at screening or current or history of clinically significant ventricular arrhythmias. Other ECG abnormalities, if considered clinically significant by the investigator **•** Participants receiving any of the prohibited medications (selected cytochrome P450 3A4 inhibitors and P450 3A4 inducers) within the specified periods or who would be likely to require prohibited concomitant therapy during the trial **•** Female participants who are breast-feeding or who have a positive pregnancy test result prior to receiving the first dose of study drug or RHEZ on day 1 **•** Current history of significant drug and/or alcohol abuse that is likely to result in poor adherence to trial requirements or that would pose a risk to the participant’s well-being during the course of the trial **•** History of current hepatitis or carriers of hepatitis B surface antigen (HBsAg) and/or anti-hepatitis C virus (HCV) **•** Participants who test positive for cocaine or other drugs of abuse (excluding known prescription stimulants and other prescribed medications and marijuana) at screening are excluded. Detectable levels of alcohol, marijuana, barbiturates, or opiates in the drug screen are not exclusionary if, in the investigator’s documented opinion, the participant does not meet *Diagnostic and Statistical Manual of Mental Disorders*, Fifth Edition criteria for moderate to severe substance use disorder and the positive test does not signal a clinical condition that would impact the safety of the participant or interpretation of the trial results, and participation is agreed to by the medical monitor prior to treatment **•** History of having taken an investigational drug within 30 days preceding trial entry **•** A history of difficulty in donating blood **•** Donation of blood or plasma within 30 days prior to dosing **•** History of serious mental disorders that, in the opinion of the investigator, would exclude the participant from participating in this trial **•** Any known prior exposure to DLM, BDQ, and QBS **•** Participants with significant medical comorbidities that in the opinion of the investigator should not participate in the trial **•** Participants with a Karnofsky score < 60 will be excluded from the trial **•** Participants testing positive for active severe acute respiratory syndrome coronavirus (SARS-CoV-2) infection at screening **•** Participants with HIV coinfection not on a stable antiretroviral regimen consisting of tenofovir, emtricitabine/lamivudine, and dolutegravir (i.e., > 3 months), or who have a detectable viral load, or who have a CD4 count < 350 cells/mm^3^ will be excluded from the trial	

*BDQ* bedaquiline, *CD4* cluster of differentiation 4 glycoprotein, *DLM* delamanid, *ECG* electrocardiogram, *HIV* human immunodeficiency virus, *QTcF* corrected QT interval using Fridericia’s method, *RHEZ* rifampicin, isoniazid, ethambutol, and pyrazinamide

Consumption of grapefruit, grapefruit products, Seville oranges, or Seville orange products within 72 h prior to the first dose of study drug (i.e., DLM, BDQ, or QBS) or RHEZ and during the trial is prohibited. Participants will be encouraged to refrain from drinking alcoholic beverages or using illicit drugs during participation in the trial.

### Who will take informed consent?

Informed consent will be freely obtained from all participants (or their guardian or legally acceptable representative, as applicable for local laws). Investigators may discuss trial availability and the possibility for entry with a potential participant without first obtaining consent. However, informed consent must be obtained and documented before initiation of any procedures that are performed solely for the purpose of determining eligibility for this trial, including withdrawal from current medication(s). Model consent form and information for participants can be found in Appendix 1.

### Interventions

#### Explanation for the choice of regimens

DLM and BDQ are two of the most recently developed anti-TB drugs that have been extensively studied in patients with TB and have received regulatory approval and World Health Organization endorsement to be used as part of an optimal background regimen for patients with TB resistant to at least rifampin [5]. DLM is a nitro-dihydro-imidazooxazole derivative that inhibits the synthesis of mycolic acid, an important component of the *M. tuberculosis* cell wall. BDQ is a diarylquinoline derivative that inhibits *M. tuberculosis* adenosine triphosphate synthase. DLM and BDQ have been studied together as part of a National Institutes of Health-sponsored combination drug-drug interaction/safety trial [14]. RHEZ tablets are a combination of four first-line agents (rifampin, isoniazid, pyrazinamide, and ethambutol) used as the standard of care in the treatment of TB worldwide [3].

#### Intervention description

For DBQ10, DBQ30, and DBQ90 treatment arms, all study drug(s) (i.e., DLM, BDQ, and QBS) will be administered after the completion of a meal. BDQ will be administered at the approved dose regimens: 400 mg daily for the first 2 weeks of treatment and 200 mg three times a week thereafter, with at least 48 h between doses for the remainder of the trial (i.e., a total of 17 weeks [DBQ10, DBQ30, and DBQ90 arms] of treatment). Study drugs will be administered with approximately 240 mL of still (noncarbonated) water. For the RHEZ treatment arm, participants will receive RHEZ orally daily for 8 weeks followed by 18 weeks of rifampin and isoniazid per the local standard of care. RHEZ will be administered with approximately 240 mL of still (noncarbonated) water and should be taken 1 h prior to or 2 h after a meal.

Although the currently approved dose for DLM is 100 mg twice daily (BID), DLM will be dosed at 300-mg QD in this study. This is based on the fact that 200-mg QD of DLM was estimated to achieve an acceptable cumulative fraction of response [15]. Following dosing of DLM 300 mg daily from previous trials, the area under the curve was about 20 to 25% higher than those obtained following 200 mg daily; correspondingly, the cumulative fraction of response also would be higher than that of the 200-mg daily dose. From a safety perspective, the exposures following 300 mg daily were lower than that following the approved 100-mg twice-daily dosing, indicating that the 300-mg daily dose would likely have less of an effect on safety outcomes [15, 16]. Once-daily dosing will provide convenience to the patients and would therefore be expected to improve compliance.

The key goal of the trial is to determine if the combination of DLM, BDQ, and QBS is efficacious, safe, and can potentially shorten the treatment duration in patients with drug-susceptible TB. To maximize the chances of success in achieving this goal, doses were chosen such that they can provide the highest possible exposures of the drugs without compromising patient safety. Three doses of QBS (10, 30, and 90 mg) were previously evaluated in a 14-day early bactericidal activity (EBA) trial in participants with TB and shown to have similar bactericidal activity, safety, and tolerability and to be similarly efficacious [12]. However, whether these doses would contribute similarly to culture conversion and subsequently a relapse-free cure is unknown. Therefore, this study will examine the three doses to confirm activity and efficacy across a wide range of doses when given for longer than 2 weeks, in a stable combination.

#### Criteria for discontinuing or modifying allocated interventions

After the first dose of the study drug(s) or RHEZ on day 1, a participant or study investigator may stop treatment permanently for a variety of reasons, including adverse events, required treatment with a disallowed medication or therapy, or treatment failure. However, each investigator must comprehensively review the circumstance and offer the participant options for continued treatment to the degree possible.

#### Strategies to ensure adherence to interventions

Study drug(s) or RHEZ will be administered using the locally accepted adherence-monitoring techniques, which could include directly observed therapy (DOT), Wisepill electronic adherence-monitoring device, video DOTs, or other adherence-monitoring techniques. In addition, all doses of study drugs given during trial visits will be observed via the hand-and-mouth procedure. Moreover, the DLM daily dosing regimen in combination with daily dosing of QBS and BDQ is being proposed in this trial due to the need to facilitate medication adherence.

#### Relevant concomitant care permitted or prohibited during the trial

The investigator will record all medications (including prescription medications, vaccines, over-the-counter medications, herbal remedies) and therapies taken by the participant from 30 days prior to signing of informed consent through the end of the evaluation period (12 months post randomization).

Quinolones are prohibited within 3 months prior to the screening visit and during the screening period. The only antiretroviral regimen allowed during this trial includes tenofovir, emtricitabine/lamivudine, and dolutegravir. Furthermore, participants must be on a stable antiretroviral regimen for at least 3 months prior to screening. The selected cytochrome P450 (CYP)3A4 inhibitors and inducers presented in Supplemental Table 2 should not be used from day -2 until the end of treatment.

Rescue treatment consisting of the standard treatment (i.e., RHEZ) will be provided to participants in the DBQ10, DBQ30, and DBQ90 treatment arms in the following circumstances: (1) If a participant is sputum culture positive for *M. tuberculosis* at the end of treatment, (2) if a participant has a microbiologic relapse subsequent to the end of treatment, or (3) if, at any time in the opinion of the investigator, a participant is clinically deteriorating, based on any combination of signs, symptoms, or chest radiograph data. Rescue treatment for participants in the RHEZ treatment arm will be individualized care according to local guidelines.

### Outcomes

A list of study outcomes can be found in Table 2.

**Table 2:** Study outcomes

*Primary*	
• To assess the safety and efficacy of quabodepistat (QBS) combined with delamanid (DLM) and bedaquiline (BDQ) in participants with drug-susceptible tuberculosis (TB) administered for 4 months compared to rifampin, isoniazid, ethambutol, and pyrazinamide (RHEZ) administered for 6 months	Safety • Adverse events (AEs), clinical laboratory tests, vital signs, physical examinations, electrocardiograms (ECGs) • The proportion of participants with a grade 3 or higher AE • The rate of all-cause treatment discontinuation Efficacy • The proportion of participants achieving sputum culture conversion ([SCC] in Mycobacteria Growth Indicator Tube [MGIT]) by the end of the treatment period
*Secondary*	
• To evaluate efficacy in sputum MGIT culture and lipoarabinomannan (LAM) for QBS combined with DLM and BDQ administered for 4 months as compared to RHEZ • To evaluate the development of resistance to anti-TB drugs in all treatment arms • To inform dose selection of QBS combined with DLM and BDQ	Efficacy • The proportion of participants who achieve SCC in MGIT by 8 weeks of treatment • Time to detection of MGIT cultures • The proportion of participants who convert sputum LAM to negative by 8 weeks of treatment and by end of treatment • Proportion of participants who develop drug resistance • Time to SCC of DLM, BDQ, and QBS as well as RHEZ
Exploratory efficacy analysis	
• To evaluate the efficacy of experimental arms in comparison to RHEZ through the end of the posttreatment follow-up period	Efficacy • The proportion of participants with favorable outcome at 12-month post-randomization • The proportion of participants with relapse at 12-month post-randomization

### Participant timeline

Each participant in this trial is expected to participate in the following periods of the trial (approximate durations listed): eligibility screening period (up 14 days); treatment period (DBQ10, DBQ30, and DBQ90 treatment arms: 17 weeks; or RHEZ treatment arm: 26 weeks); and the post-treatment follow-up period (12 months post randomization). Overall, the per-patient trial duration from signing of the first informed consent form to the final participant assessment is expected to be approximately 12 months. In total, there will be 27 visits; see Table 3 for a schedule of selected assessments.

**Table 3:** Schedule of selected assessments

Period	Pre-treatment period		Treatment period																		Follow-up period						
Visit	1	2	3	4	5	6	7	8	9	10	11	12	13	14	15	16	17	18	19	20	21	22	23	24	25	26	27
	Day		Week (±2 days)																		Week (±7 days)						
Day/week	−14 to −2	−1	1	1	2	3	4	5	6	7	8	10	12	13	15	17	19	21	23	26	28	30	34	39	43	47	52
Informed consent	X																										
Demographics and medical history^a^	X																										
Concomitant medication	X	X	X	X	X	X	X	X	X	X	X	X	X	X	X	X	X	X	X	X	X	X	X	X	X	X	X
Inclusion/exclusion	X	X																									
Randomization		X																									
Chest radiograph^b^	X															X				X							X
PET/CT scan^c^		X					X									X				X							
Standard 12-lead ECG	X	X			X		X		X		X	X	X	X	X	X		X		X		X	X	X	X	X	X
Signs and symptoms of TB	X	X			X		X		X		X			X		X		X		X		X	X	X	X	X	X
Hematology, serum, chemistry, and urinalysis	X	X			X		X		X		X			X		X		X		X							X
Sputum MGIT Culture/sputum LAM and RS ratio^d^		X	X	X	X	X	X	X	X	X	X	X	X	X	X	X	X	X	X	X	X	X	X	X	X	X	X
Study drug administration			X	X	X	X	X	X	X	X	X	X	X	X	X	X	X	X	X	X	X						
AEs and IREs	X	X	X	X	X	X	X	X	X	X	X	X	X	X	X	X	X	X	X	X	X	X	X	X	X	X	X

^a^Includes TB treatment history. ^b^An end-of-treatment chest radiograph will be taken at week 17 (for participants in DBQ10, DBQ30, and DBQ90 treatment arms) or week 26 (for participants in the RHEZ treatment arm) and at month 12. ^c^Only for participants consenting to this trial procedure. The PET/CT scan will be done fasting within 1 week prior to starting treatment, week 4 (±7 days), and at the end of treatment visit (±7 days) for each arm (i.e., week 17 for DBQ10, DBQ30, and DBQ90 treatment arms and week 26 for the RHEZ treatment arm). ^d^If the participant is willing and able to spontaneously produce a subsequent specimen during the visit, specimen number 3 will be collected directly into ribonucleic acid preservation media and frozen for batch measuring of the RS ratio. If this sputum cannot be collected, this will not be considered a protocol deviation. *AE* adverse event, *DBQ* delamanid+bedaquiline+qubodepistatat, *ECG* electrocardiogram, *IRE* immediately reportable event, *LAM* lipoarabinomannan, *MGIT* Mycobacteria Growth Indicator Tube, *PET/CT* positron emission tomography-computed tomography, *RHEZ* rifampicin, isoniazid, ethambutol, and pyrazinamide, *RS* ribosomal ribonucleic acid synthesis, *TB* tuberculosis

### Sample size

It is planned that enough participants will be enrolled so that there will be 20 participants in DBQ10, 40 participants each in DBQ30 and DBQ90, and 20 participants in the RHEZ arm. This is a pilot trial and exploratory in nature; thus, 80% confidence intervals (CIs) for difference in sputum culture conversion (SCC) will be calculated to compare with a noninferiority margin. Assuming that the SCC rate for the primary endpoint is 93% for the RHEZ arm—and assuming the SCC rate of the investigational arms is identical to the RHEZ arm—then with a noninferiority margin of 12%, a sample size of 120 participants (100 for pooled DBQ arms and 20 for RHEZ) will have about 84% power to reject the null hypothesis with a one-sided alpha level of 0.1; i.e., the lower limit of the two-sided 80% CI of the difference in the rates of sputum culture conversion between the pooled investigational arms and RHEZ is larger than −0.12. The Miettinen and Nurminen method to construct 80% CI was used in the sample size calculation [17]. Assuming 11% of randomized participants will not demonstrate at least 1 positive culture result by week 1 and susceptibility to rifampin and isoniazid, approximately 135 participants will be randomized in a 1:2:2:1 ratio to obtain 120 participants in the modified intent-to-treat analysis set.

### Assignment of interventions: allocation

#### Sequence generation

Treatment allocation will be performed using a stratified block randomization technique. Stratification will be by HIV status (yes/no) and the presence of bilateral cavitations (yes/no) on the screening chest x-ray. A separate block randomization list will be generated for each of the four strata. Randomization numbers generated within a block can be allocated to only one site. The ratio of treatment allocation to DBQ10, DBQ30, DBQ90, and RHEZ, within each block (and consequently, overall), will be 1:2:2:1, respectively. The PROC PLAN procedure in Statistical Analysis System (SAS) v9.4 will be used to generate the randomization numbers and treatment allocations for this study.

#### Concealment mechanism

As specified in the previous section, treatment allocations will be made within blocks. Block size will be blinded for all personnel involved in the trial and will only be revealed at the time of the final database lock. In addition, to avoid bias in assessment of the primary endpoint of sputum culture conversion, the microbiology laboratory analyzing sputum culture will be blinded to the treatment assignment.

#### Implementation

An independent statistician will generate the randomization lists to be used in the trial. Trial personnel will not have access to the randomization lists during the recruitment phase to ensure unbiased treatment assignment at sites. For recruiting participants, newly diagnosed drug-susceptible pulmonary TB participants will be identified at treatment clinics, and their interest in the trial will be gauged. The recruitment period will be for 1 year, with an estimated recruitment rate of 3–4 patients/site screened/month, with an enrollment rate of 40% per site.

### Assignment of interventions: blinding

#### Who will be blinded?

This is an open-label trial. Once issued, treatment assignments will not be blinded. To avoid bias in assessment of the primary efficacy endpoint, the microbiology laboratory will be blinded to treatment assignments.

### Data collection and management

#### Plans for assessment and collection of outcomes

During each participant’s visit to the site, an investigator or their designee participating in the trial will record information to document all significant observations. At a minimum, these notes will contain the following: documentation of the informed consent process, including any revised consents; documentation of the investigator’s decision to enroll the participant into the trial, the review of all inclusion/exclusion criteria prior to study drug administration, and confirmation of the participant’s actual participation in the trial; the date of the visit and the corresponding visit or day in the trial schedule; and general participant status remarks, including any significant medical findings. The severity, frequency, duration, action taken, and outcome of any adverse events (AEs) and the investigator’s assessment of relationship to study drug(s) must also be recorded, including any changes in concomitant medications or dosages; a general reference to the procedures completed, including dosing and study drug compliance; and the signature (or initials) and date of the investigator (or designee) who made an entry in the medical record. Any contact with the participant via telephone or other means that provide significant clinical information will also be documented in the progress notes as described above.

#### Plans to promote participant retention and complete follow-up

For participants who cannot be contacted on or before the trial visits at week 17 (for DBQ10, DBQ30, and DBQ90 treatment arms) and week 26 (for RHEZ treatment arm) during the treatment period and/or who do not have a known reason for discontinuation (e.g., AE or withdrew consent), the site will make three documented attempts to contact the participant by telephone; in the event the site is unable to reach the participant by telephone, the site will attempt to contact the participant via certified mail or an alternative similar method, where appropriate, before assigning a “lost to follow-up” status. Participants will be encouraged to remain in the study even if they wish to discontinue treatment.

### Data management

Information from medical records and other source documents will be entered by investigative site personnel onto electronic case report forms (eCRFs) in the sponsor’s electronic data capture (EDC) system that is 21 Code of Federal Regulations Part 11 compliant. Changes to the data will be captured by an automatic audit trail in the EDC system. Electronic data not entered on eCRF, such as data received from central laboratories and central electrocardiogram (ECG) readers, will be reconciled using key data fields by the sponsor or the clinical research organization with the eCRF data to ensure consistency.

The investigator will ensure that the trial site file is maintained in accordance with applicable International Council for Harmonisation Good Clinical Practice guidance and as required by applicable local regulations. The investigator/institution will take measures to prevent accidental or premature destruction of these documents.

### Confidentiality

All information generated in this trial will be considered confidential and will not be disclosed to anyone not directly concerned with the trial without the sponsor’s prior written permission. Subject confidentiality requirements of the region(s) where the trial is conducted will be met. However, authorized regulatory officials and sponsor personnel (or their representatives) may be allowed full access to inspect and copy the records, consistent with local requirements.

#### Plans for collection, laboratory evaluation, and storage of biological specimens for genetic or molecular analysis in this trial/future use

All study drugs, participant bodily fluids, and/or other materials collected shall be used solely in accordance with this protocol, unless otherwise agreed to in writing by the sponsor. Participants will be identified only by unique participant ID in the eCRF. If further participant ID is required, participants’ full names may be made known to a regulatory agency or other authorized officials if necessary.

### Statistical methods

#### Statistical methods for primary and secondary outcomes

The primary efficacy endpoint of the trial is the proportion of participants who have SCC and no subsequent positive culture by the end of treatment. Sputum culture conversion occurs when a participant has the first of two visits ≥ 1 week apart with sputum cultures negative and without a positive sputum culture result in between. The three DBQ arms will be pooled, and noninferiority with RHEZ will be established if the lower limit of 80% CI for the difference of proportion of participants with SCC between the pooled DBQ arm is larger than −0.12. The Miettinen and Nurminen CI of common risk difference stratified by HIV status and cavitation status prior to randomization will be used [17]. If there is a stratum of very small size, strata may be collapsed.

The secondary efficacy endpoints of this protocol are the proportion of participants in each arm who achieve SCC in Mycobactera Growth Indicator Tube (MGIT) by 8 weeks of treatment, time to detection of MGIT cultures, the proportion of participants in each arm who convert sputum lipoarabinomannan (LAM; a biomarker of bacterial load) to negative by 8 weeks of treatment and by end of treatment as determined using the Otsuka LAM-enzyme-linked immunosorbent assay (ELISA) to quantitate LAM concentration [18], proportion of participants who develop drug resistance, and time to SCC. For a proportion endpoint, the same analysis methodology specified for the primary endpoint will be used. A 12% noninferiority margin will be used if the goal is to establish noninferiority. The Kaplan-Meier curve of time to SCC will be provided for each arm, and relationship between time to SCC and pharmacokinetic parameters will be studied. Control of experiment-wise type 1 error is not applicable to this trial.

### Interim or additional analyses

Interim pharmacokinetic/pharmacodynamics analyses will be performed after participants have completed the end of treatment response time point (4 months of treatment in the DBQ arms). In addition, an early assessment of dose response and toxicity may be conducted after the first 24 randomized participants (i.e., approximately 4:8:8:4 in each arm) complete the end of treatment response time point. If a DBQ arm is dropped, subsequently enrolled participants would then be allocated to the remaining DBQ arms. Other interim analyses may be performed for internal development purposes. The interim analysis will not result in stopping of the study unless it is due to safety concerns. No statistical comparisons will be performed at the interim; hence, no adjustments for multiple testing need to be made in this study.

### Methods in analysis to handle protocol non-adherence and any statistical methods to handle missing data

Participants who discontinue early from the trial during the treatment period without an SCC outcome will be considered as not having achieved SCC.

### Plans to give access to the full protocol, participant level-data, and statistical code

To submit inquiries related to Otsuka clinical research, or to request access to individual participant data (IPD) associated with any Otsuka clinical trial, please visit https://clinical-trials.otsuka.com/. For all approved IPD access requests, Otsuka will share anonymized IPD on a remotely accessible data sharing platform.

### Oversight and monitoring

#### Composition of the coordinating center and trial steering committee

The trial is not supported by an external steering committee nor a stakeholder and public involvement group (SPIG). Instead, a cross-functional project team within Otsuka Pharmaceutical Development & Commercialization designed the study and is responsible for the day-to-day operational execution of the trial along with support from a clinical research organization (CRO) team. A national principal investigator (PI) was appointed to provide additional support. In this role, the national PI assumes overall responsibility for the conduct of the trial, coordinates concerns of all investigators regarding the conduct of the trial, and communicates with Otsuka Pharmaceutical Development & Commercialization, ethics committees, and the regulatory authority as necessary.

#### Composition of the data monitoring committee, its role, and reporting structure

An independent Data Monitoring Committee (DMC) will be contracted by the sponsor to review safety and efficacy data during the conduct of the trial. The DMC will convene and operate based on an established charter. The DMC will be permitted to seek additional independent expertise as needed. The primary responsibility of the DMC will be to monitor safety and to advise the sponsor of the clinical project regarding whether the safety concerns merit stopping the trial. Toward this objective, the DMC will meet on a predetermined schedule to review safety and available efficacy data.

### Adverse event reporting and harms

The investigator will regularly assess participants for the occurrence of AEs. To avoid bias in eliciting AEs, participants will be asked the nonleading question: “How have you felt since your last visit?” All AEs (serious and nonserious) reported by the participant will be recorded on the source documents and eCRF provided by the sponsor. Adverse event collection will begin after a participant signs the informed consent form and will continue until the follow-up visit 12 months post randomization. All AEs will be reported after participant informed consent has been obtained, including screening failures due to AEs, irrespective of study drug administration.

Medical terminology will be used for AE reporting. Adverse events will be reported as a single unifying diagnosis whenever possible or, in the absence of a unifying diagnosis, as individual signs or symptoms. An insurance policy is in place if needed for any who would suffer harm from trial participation.

### Frequency and plans for auditing trial conduct

The sponsor’s quality assurance unit (or representative) may conduct trial site audits. Audits will include, but are not limited to, study drug supply, the presence of required documents, the informed consent process, site operations, delegation of authority and training, and a review of the eCRF with source documents, as applicable. The investigator will agree to cooperate and participate with audits. Regulatory authorities may inspect the investigator site during or after the trial. The investigator will cooperate with such inspections and will contact the sponsor immediately if such an inspection occurs.

#### Plans for communicating important protocol amendments to relevant parties (e.g., trial participants, ethical committees)

The investigator will not make any changes to the protocol without the sponsor’s prior written consent and subsequent approval/favorable opinion by the IRB/independent ethics committee (IEC). Any permanent change to the protocol, whether an overall change or a change for a specific trial site(s), must be handled as a protocol amendment. Any amendment will be written by the sponsor. Each amendment will be submitted to the IRB/IEC, as required by local regulations. Except for “administrative” or “nonsubstantial” amendments, investigators will wait for IRB/IEC approval/favorable opinion of the amended protocol before implementing the change(s). Administrative amendments are defined as having no effect on the safety of participants, conduct or management of the trial, trial design, or the quality or safety of study drug(s) used in the trial. A protocol change intended to eliminate an apparent immediate hazard to participants should be implemented immediately after agreement by the sponsor and investigator, followed by IRB/IEC notification within local applicable timelines. The sponsor will submit protocol amendments to the applicable regulatory agencies within local applicable timelines.

When the IRB/IEC, investigators, and/or the sponsor conclude that the protocol amendment substantially alters the trial design and/or increases the potential risk to the participant, the currently approved written informed consent form will require similar modification. In such cases, after approval/favorable opinion of the new informed consent form by the IRB/IEC, repeat written informed consent will be obtained from participants enrolled in the trial before expecting continued participation and before the amendment-specified changes in the trial are implemented.

### Dissemination plans

The results of this study will be disseminated through peer-reviewed journals, conference presentations, and social media.

## Discussion

There remains a need for new, safe, and effective anti-TB agents that can be used in combination with available agents to shorten the duration of anti-TB therapy and prevent the development of resistance. The advent of newer, more efficacious regimens could help optimize TB therapy, enhance compliance, and potentially improve outcomes. Although the need for new agents and regimens is clear, the most efficient way to evaluate regimens is still in evolution. Traditionally, in order to establish efficacy, a new TB compound will first be examined in a 14-day EBA trial, as done for QBS. The next step is a 2-month SCC trial, in which TB patients are treated for 2 months with a regimen containing the new compound or added to a standard of care and then continue the treatment on standard of care to complete the treatment. The regimen containing the new compound can then go into a phase 3 trial. The challenge in this approach is that the selection of regimens for the phase 3 trials is only based on the 2-month SCC, which may or may not translate to cure. Our trial design allows the evaluation of potential cure in the phase 2 stage that is likely to provide better evidence to guide the phase 3 regimen selection. Our preclinical and available clinical data for QBS alone and in combination with DLM and BDQ provide the strong basis for the 4-month treatment; thus, this study is likely to be of great interest to the public health community.

This phase 2b/c, open-label, randomized, dose-finding trial is designed to evaluate the safety, tolerability, and efficacy of QBS in combination with delamanid and/or BDQ compared to RHEZ (the normal standard of care) in participants with newly diagnosed pulmonary drug-sensitive TB. While this study is only 120 participants and open label, it is designed to provide information that will facilitate further development in a larger population.

The range of QBS doses proposed for this study (10, 30, and 90 mg) was selected to completely characterize the entire exposure-response spectrum and to gain data that will allow future dosage recommendations. Moreover, dose finding is usually done by using 2-month culture conversion as an endpoint; however, this trial was designed to bypass this by using a 4-month endpoint. This approach may shorten the development timelines. The dose of delamanid selected for use in DBQ10, DBQ30, and DBQ90 arms (300 mg) was chosen based on the observation that the EBA of delamanid in adults with newly diagnosed smear-positive pulmonary TB appears to plateau at this level (regimens studied in the referenced EBA trial were 100 mg, 200 mg, 300 mg, or 400 mg once daily for 14 days) [15]. The study will include standard treatment as an active comparator. Efficacy in this study will be determined by the proportion of participants who have SCC and no subsequent positive culture by the end of treatment.

The results from this trial will determine if 4 months of combination therapy with QBS are tolerable and effective to treat patients with newly diagnosed smear-positive pulmonary TB.

Patient recruitment began April 12, 2022, and is expected to end in February 2024.

### Supplementary Information

**Additional file 1: Supplementary Table 1.** Study sites. **Supplemental Table 2.** List of selected cytochrome P450 3A4 inhibitors and cytochrome P450 3A4 inducers prohibited during the trial.** Appendix 1:** Participant information and informed consent form.

**Additional file 1: **SPIRIT Checklist for *Trials.*

## Data Availability

To submit inquiries related to Otsuka clinical research or to request access to individual participant data (IPD) associated with any Otsuka clinical trial, please visit https://clinical-trials.otsuka.com/. For all approved IPD access requests, Otsuka will share anonymized IPD on a remotely accessible data sharing platform.

## Abbreviations

AEs: Adverse events
BDQ: Bedaquiline
BID: Twice daily
CIs: Confidence intervals
CRO: Clinical Research Organization
CYP: Cytochrome
DBQ10: DLM (300 mg once daily [QD]) + BDQ (400 mg QD × 2 weeks and then 200 mg thrice weekly [TIW]) + QBS (10 mg QD) for 17 weeks
DBQ30: DLM (300 mg QD) + BDQ (400 mg QD × 2 weeks and then 200 mg TIW) + QBS (30 mg QD) for 17 weeks
DBQ90: DLM (300 mg QD) + BDQ (400 mg QD × 2 weeks and then 200 mg TIW) + QBS (90 mg QD) for 17 weeks
DLM: Delamanid
DMC: Data Monitoring Committee
DOT: Directly observed therapy
DprE1: Decaprenylphosphoryl-β-D-ribose-2′-oxidase
EBA: Early bactericidal activity
ECG: Electrocardiogram
eCRFs: Electronic case report forms
EDC: Electronic Data Capture
ELISA: Enzyme-linked immunosorbent assay
HIV: Human immunodeficiency virus
IPD: Individual participant data
IRB/IEC: Institutional Review Board/Independent Ethics Committee
LAM: Lipoarabinomannan
MGIT: Mycobactera Growth Indicator Tube
PI: Principal investigator
QBS: Quabodepistat
QD: Once daily
RHEZ: Rifampicin, isoniazid, ethambutol, and pyrazinamide
SAS: Statistical Analysis System
SCC: Sputum culture conversion
SPIG: Stakeholder and public involvement group
TB: Tuberculosis
TIW: Thrice weekly
