# Supplementary material for: Quabodepistat in combination with delamanid and bedaquiline in participants with drug-susceptible pulmonary tuberculosis: protocol for a multicenter, phase 2b/c, open-label, randomized, dose-finding trial to evaluate safety and efficacy
Source: Trials. 2024 Jan 19;25:70. doi: 10.1186/s13063-024-07912-5 (PMC10799444; doi:10.1186/s13063-024-07912-5)
Supplement: Supplementary file 1 — Additional file 1: Supplementary Table 1. Study sites. Supplemental Table 2. List of selected cytochrome P450 3A4 inhibitors and cytochrome P450 3A4 inducers prohibited during the trial. Appendix 1: Participant information and informed consent form. [file 13063_2024_7912_MOESM1_ESM.pdf]

SUPPLEMENTARY MATERIAL

Supplementary Table 1. Study sites

**Study Sites**
Aurum Institute – Tembisa Clinical Research Centre<br>Tembisa, Gauteng, South Africa, 1632
Aurum Rustenburg<br>Rustenburg, South Africa, 2999
TASK Brooklyn Chest Hospital<br>Cape Town, South Africa, 7100
University of Cape Town Lung Center Institute<br>Cape Town, South Africa, 7700
Masiphumelele Clinical Research Site<br>Cape Town, South Africa, 7975
Themba Lethu Clinic Clinical HIV Research Unit (CHRU)<br>Johannesburg, South Africa, 2092
Perinatal HIV Research Unit Tshepong Hospital Complex<br>Klerksdorp, South Africa, 2574
Setshaba Research Center<br>Pretoria, South Africa, 0152

Supplemental Table 2. List of selected cytochrome P450 3A4 inhibitors and cytochrome P450 3A4 inducers prohibited during the trial

**Potent CYP3A4 Inhibitors**	
Viekira PAK2<br>Cobicistat<br>Ketoconazole<br>Troleandomycin<br>Telaprevir<br>Posaconazole<br>Telithromycin<br>Grapefruit juice<br>Conivaptan<br>Tipranavir	Itraconazole<br>Mibefradil<br>Clarithromycin<br>Nelfinavir<br>Nefazodone<br>Idelalisib<br>Boceprevir<br>Voriconazole<br>Ritonavir
**Moderate CYP3A4 Inhibitors**	
Erythromycin<br>Fluconazole<br>Atazanavir<br>Diltiazem<br>Darunavir<br>Dronedarone<br>Crizotinib<br>Atazanavir<br>Aprepitant<br>Casopitant<br>Amprenavir	Faldaprevir<br>Imatinib<br>Verapamil<br>Netupitant<br>Nilotinib<br>Tofisopam<br>Cyclosporine<br>Ciprofloxacin<br>Isavuconazole<br>Cimetidine
**Potent CYP3A4 Inducers**	
Carbamazepine<br>Mitotane<br>St. John's wort	Enzalutamide<br>Phenytoin
**Moderate CYP3A4 Inducers**	
Bosentan<br>Etravirine	Efavirenz<br>Modafinil

PARTICIPANT INFORMATION AND INFORMED CONSENT FORM

**Protocol no:**	323-201-00006
**Protocol title:**	A Multicenter, Phase 2b/c, Open-label, Randomized, Dose-finding Trial to Evaluate the Safety and Efficacy of a 4-month Regimen of OPC-167832 in Combination with Delamanid and Bedaquiline in Participants with Drug-susceptible Pulmonary Tuberculosis in Comparison with Standard Treatment
**Sponsor:**	Otsuka Pharmaceutical Development & Commercialization, Inc.<br>2440 Research Blvd.<br>Rockville, MD 20850, United States
**Participant no:**	*<Add Participant No.>*
**Investigator:**	*<Add Investigator name, address, and telephone number>*
**Version:**	**5.0**

Dear Participant,

You are invited to participate in a research study with OPC-167832, Delamanid and Bedaquiline as investigational medications. An investigational medication is a medication that is approved by regulatory authorities (such as the US FDA, SAHPRA) for testing in humans but has not yet been approved and registered for routine use.
Before you agree to take part in this research study, you must please read this Participant Information and Informed Consent Form as it contains important information to help you decide if it is in your best interest.

You are encouraged to ask as many questions as needed to make sure you understand the study procedures, including possible risks and benefits. If you have any questions that are not properly answered in this Participant Information and Informed Consent Form, please ask a study staff member to give you more information. You are welcome to take this document home with you and discuss the study with your family and friends.

<Add name of Ethics Committee > and the SAHPRA have approved the study for compliance with medical and ethical standards. In addition, the study will follow the 2013 Declaration of Helsinki and The South African Good Clinical Practice: Clinical Trial Guidelines third edition, 2020, which describes your rights as a research participant. These guidelines will guide the study doctor (investigator) in biomedical research involving human participants.

The study doctor will be paid by the Sponsor to conduct this study.

1. Your rights as a participant

Your doctor will determine if you are able to join this study. If you decide to join, you will be asked to sign this consent form. By signing this form, you agree to follow the instructions given by the research staff during the study. Your participation in this study is voluntary. You may choose not to be in the study or to leave the study at any time by telling the study doctor. If you decide not to participate in the study or to

withdraw your consent, you will not lose any benefits to which you are otherwise entitled. If any changes are made during that study that affect any of the study procedures or information provided to you in this informed consent form, you will be informed by the study doctors and asked to review and sign an updated informed consent form.

The study doctor has the right to withdraw you from the study if it is in your best interest, in which event, the reason(s) will be given to you.

If you did not provide the correct medical history or did not follow the guidelines in this document and the instruction of the investigator pr site staff, you may be withdrawn from the study at any time.

2. Purpose of the study

The purpose of this study is to find out how safe and effective OPC-167832 is when given with Delamanid and Bedaquiline (BDQ) for 17 weeks, compared to the local standard of care medicine given for tuberculosis (TB). The standard of care medicine is Rifafour®, which is a combination of four medicines: Rifampicin, Isoniazid, Ethambutol and Pyrazinamide, given for 8 weeks, followed by Rifampicin and Isoniazid, given for 18 weeks. The standard of care medicine is given for a total of 26 weeks (6 months).

Approximately 120 participants will be enrolled in the study. There are about 7 sites in South Africa participating in this study.

3. Study design

You will be on the study for about 1 year (52 weeks). This includes the pre-treatment period, treatment period and follow-up visits.

- Pre-treatment period: Includes 2 visits to the trial site for screening and baseline reviews where several tests and clinical examinations will be done.
- Treatment period: May last for 17 weeks or 26 weeks, depending on the treatment you have been assigned.
- You will be expected to go to the trial site every week for the first 8 weeks (up to Visit 11) then
    - every 2 weeks for 1 month (Visits 12 and 13), then
    - one week later for Visit 14,
    - then every 2 weeks till Visit 19.
    - Depending on your treatment you may attend a visit 3 weeks later (Visit 20).
- Follow-up visits start 2 - 5 weeks later and visits are every 2 weeks till Visit 22,
    - then monthly for 5 months (Visit 23 – 27).
    - You finish the study at Visit 27, approximately 12 months after you start treatment.

There are 4 treatment groups in this study. You will be randomly assigned (like flipping a coin) to receive 1 of the 4:

- Delamanid (300 mg given once a day) and BDQ (400 mg given once a day for 2 weeks, then 200 mg three times a week and OPC-167832 (10 mg given once a day) for 17 weeks
- Delamanid (300 mg given once a day) and BDQ (400 mg given once a day for 2 weeks, then 200 mg three times a week and OPC-167832 (30 mg given once a day) for 17 weeks
- Delamanid (300 mg given once a day) and BDQ (400 mg given once a day for 2 weeks, then 200 mg three times a week and OPC-167832) (90 mg given once a day) for 17 weeks
- Rifafour is given for 8 weeks followed by 18 weeks of rifampin and isoniazid (this is 26 weeks)

You cannot choose which treatment group you are assigned. Your study doctor will tell you which treatment group you have been randomly assigned to.

OPC-167832, Delamanid and BDQ will be taken with a full glass of still water after you have finished a meal (breakfast)

If you are assigned to Rifafour, this will be taken one hour before a meal (breakfast) or 2 hours after a meal (breakfast) with a full glass of still water.

To make it easier to understand the procedures explained below we have called OPC-167832, Delamanid, BDQ and Rifafour 'study treatment'.

Your medication will be given to you in a small medication box called WisePill that you will take home and bring back at every visit to the trial site. The medicine box makes a sound at a set time every day to remind you to take your medication and allows you to open the medicine box for that day. Further instructions on how the medicine box is used will be given to you when you get your medication.

It is important to ensure that you receive and take all study TB medicines and complete study treatment as required by the study. This is very important in order to monitor your response to treatment. Taking many different tablets each day can be difficult. We have put different methods in place to help you take the study medications as required in the study such as Direct Observed Therapy (DOT) and regular phone calls.
Directly observed therapy (DOT) is done when it is very important that a person takes every dose of medicine. DOT is a method of taking your tablets in which study staff or somebody that you have chosen will watch as you take each dose of medication. DOT may take place five times a week, at a suitable time as set on your medication box alarm.

If you cannot do DOT or do not agree to allow a study staff member or somebody that you choose to watch you take your dose of medication, then a study staff member will call you five times a week to check if you have taken your study treatment according to study requirements.

4. Study procedures

All your visits will be outpatient (clinic) visits.
Tests and procedures, called safety assessments, will be done on various visits.
The chart below gives all the tests and procedures that will be done during the study and the days that they will be done. These are also explained on the pages below the charts. All tests and procedures are done to monitor your safety.

If you are assigned to Rifafour, you will complete all visits.

	Pre-Treatment Period		Treatment Period																		Follow up period							
**Visit**	1 Screening	2 Baseline	3	4	5	6	7	8	9	10	11	12	13	14	15	16	17	18	19	20	21	22	23	24	25	26	27	ET
	Day			Week (± 2 days)																			Week (± 7 days)					
**Day/ Week**	−14 to −2	−1	1	1	2	3	4	5	6	7	8	10	12	13	15	17	19	21	23	26	28	30	34	39	43	47	52	N/A
Informed consent *	X																											
Demographic information	X																											
Medical history and past TB treatment if any	X																											
Review to see if you can be included	X	X																										
Chest X ray**	X															X				X							X	X
Randomization		X																										
Complete Physical examination	X																										X	
Targeted physical examination		X			X		X		X		X	X	X	X	X	X		X		X		X	X	X	X	X		X
Karnofsky Score	X																											
Vital signs (Lying down)	X Lying down then sitting	X			X		X		X		X	X	X	X	X	X		X		X	X	X	X	X	X	X	X	X
Eye Test **	X															X				X								
PET / CT scan**		X					X									X				X								
ECG	X	X			X		X		X		X	X	X	X	X	X		X		X		X	X	X	X	X	X	X
Signs and symptoms of TB	X	X			X		X		X		X			X		X		X		X		X	X	X	X	X	X	X

	Pre-Treatment Period		Treatment Period																		Follow up period							
**Visit**	**1** Screening	**2** Baseline	**3**	**4**	**5**	**6**	**7**	**8**	**9**	**10**	**11**	**12**	**13**	**14**	**15**	**16**	**17**	**18**	**19**	**20**	**21**	**22**	**23**	**24**	**25**	**26**	**27**	**ET**
	**Day**			**Week (± 2 days)**																			**Week (± 7 days)**					
**Day/ Week**	**−14 to −2**	**−1**	**1**	**1**	**2**	**3**	**4**	**5**	**6**	**7**	**8**	**10**	**12**	**13**	**15**	**17**	**19**	**21**	**23**	**26**	**28**	**30**	**34**	**39**	**43**	**47**	**52**	**N/A**
Blood for Haematology, chemistry and urine collected for urinalysis	X	X			X		X		X		X			X		X		X		X							X	X
Blood collected for clotting	X																											
Blood for HIV and Hepatitis	X																											
Blood for HbA1c (to check sugar level over past 2-3 months)	X																											
Blood for CD4 and HIV viral load only if you have HIV	X																			X							X	X
Blood for gene testing (optional)	X	X			X		X		X		X			X		X		X		X							X	X
Blood for PK (drug levels) of OPC-167832, Delamanid, DM-6705 and BDQ (and M2)			X 2 times		X Once		X Once				X 2 times			X Once		X 2 times												X 2 times
Urine drug screen	X																											
Urine pregnancy test	X																										X	X
Test for COVID	X																											
Sputum collection (Number of samples collected)	X	X	X	X	X	X	X	X	X	X	X	X	X	X	X	X	X	X	X	X	X	X	X	X	X	X	X	X

	Pre-Treatment Period		Treatment Period																		Follow up period							
**Visit**	1 Screening	2 Baseline	3	4	5	6	7	8	9	10	11	12	13	14	15	16	17	18	19	20	21	22	23	24	25	26	27	ET
	Day			Week (± 2 days)																			Week (± 7 days)					
**Day/ Week**	−14 to −2	−1	1	1	2	3	4	5	6	7	8	10	12	13	15	17	19	21	23	26	28	30	34	39	43	47	52	N/A
Acid Fast Bacilli Smear Microscopy		X																										
Antibiotic Susceptibility Testing (testing of your TB bacteria to check that the Tb medication is working against them)		X	X														X									X		
OPC-167832, Delamanid and BDQ if assigned			X	X	X	X	X	X	X	X	X	X	X	X	X	X												
Check that OPC-167832, Delamanid and BDQ was taken correctly			X	X	X	X	X	X	X	X	X	X	X	X	X	X												
RHEZ if assigned			X	X	X	X	X	X	X	X	X	X	X	X	X	X	X	X	X	X	X							
Check that RHEZ was taken correctly			X	X	X	X	X	X	X	X	X	X	X	X	X	X	X	X	X	X	X							
Doctor checks for any adverse events	X	X	X	X	X	X	X	X	X	X	X	X	X	X	X	X	X	X	X	X	X	X	X	X	X	X	X	X
Doctor checks all medications	X	X	X	X	X	X	X	X	X	X	X	X	X	X	X	X	X	X	X	X	X	X	X	X	X	X	X	X

***Informed Consent:** At this first visit the study doctor will discuss the information in this informed consent form and will also tell you about the HIV consent form, Genomics consent form, PET scan consent form and give you information about the Sub Study.

**** The Week 17 chest xray, PET/CT scan and eye test will only be performed if you are assigned to take OPC-167832, Delamanid and Bedaquiline treatments. The Week 26 chest xray, PET/CT scan and eye test will only be performed if you are assigned to take RHEZ treatment.

The procedures noted in the chart above are described in the section below

Demographic information	This is your age, date of birth, race, gender and marital status. You will also need to give your address and contact numbers.
Medical History	Please tell the study doctor of all illnesses you have had as well as any operations or reasons you were admitted to hospital. Please also tell the doctor if you have had TB in the past and if you took medicine for TB.<br>You will also be asked to tell the study doctor ALL the medicines, vitamins or herbal medications you have taken during the last 30 days, including any vaccines you may have received.
Physical examination<br>and<br>targeted examination	The study doctor will examine your head, ears, eyes, nose, and throat; neck and chest; abdomen; arms and legs; nervous system; and skin; and look inside of your mouth. He/she will ask you questions about how you feel. This is a full physical examination and will be done twice (at the first visit and on the last day of the study.)<br>When you start your study treatment, the study doctor will only check parts of your body if you have any complaints, pains or disturbances and this may lead to further examination of the problematic area. For example, if you have a cough, he will listen to your chest. At a minimum, your study doctor will check general appearance and listen to your chest. This is called the targeted examination and will be done 19 times during the study.
Eye Test	An eye test will be done two times during the study to check how well you see the details of the letters or symbols on a chart from a distance and also a test for change in color vision testing. These tests may be done again during the study if the study doctor finds anything wrong or if you develop any eye problems during the study. If you are assigned to OPC-167832, Delamanid and BDQ you will have an eye test at screening and at week 17. If you are assigned to Rifafour you will have an eye test at screening and at week 26.
Chest X-ray	A chest X-ray is a photograph of your chest made by using radiation. It will be done at the screening visit, week 17, week 26 and at the end of the study. If you are assigned to OPC-167832, Delamanid and BDQ you will also have a chest X-ray at week 17. If you are assigned to Rifafour you will have a chest X-ray at screening and week 26.
PET / CT Scan	A PET/CT scan is a special type of image (picture)t that helps doctors identify diseases, such as some infections or cancer. Before the scan you are injected with FDG, a type of sugar that gives off a small amount of radiation. The PET machine can detect and map out the areas in your lungs that take up the FDG because they are more active than other areas. The CT machine uses x-rays to take pictures of tissues and organs inside the body. Together, the PET and CT images give a good picture of the structure and activity of your lungs. The PET/CT scanner is at another location and < name of site> staff will drive you there and stay with you until it is time to come home. This visit will take most of the day. You will be asked to sign a separate informed consent form with more details about the PET / CT Scan.<br>The PET/ CT Scan will be done 3 times during the study. The first time will be within a week before starting the study treatment, at week 4 and at the end of your study treatment.
Blood tests	Blood, about 15ml (3 teaspoons), will be used to test chemistry, and haematology at each visit. These blood tests show how well your body is working and reacting to the treatment and will be done 11 times during the study.<br>At the first visit, another 17ml (3 ½ teaspoons) of blood will also be taken to check blood clotting, alcohol levels and Hepatitis infection. A total of about 32 ml (6 ½ teaspoons) will be collected on the first visit.

	You will also be required to have an HIV test. A separate consent form will be provided for you to review before having the HIV Test. If you know you are HIV-infected or are found to be HIV-infected, you will be required to have a CD4 count (test which determines the number of cells in your blood that fight against HIV) and a HIV viral load (a test that measure the amount of HIV in your blood).
Urine tests	You will need to provide a urine sample, about 100ml (about ½ a cup) in a sampling cup. A test will be done on your urine to check how healthy you are.<br>Tests will also be done for drugs (drugs of abuse), at the beginning of the study.<br>If you are female, your urine will also be tested to check if you are pregnant.
COVID test	A swab will be inserted either into the back of your throat or up your nostril to obtain mucous to test for COVID-19 infection. You may not join the study if you test positive for COVID at your first visit.
Vital signs	Your blood pressure, heart rate, temperature, breathing rate when you are lying down, and your weight will be checked 20 times during the study.<br>At your first visit, your blood pressure and heart rate will be taken when you are lying down and when you are sitting. You must be in each position for at least 3 minutes before your blood pressure and heart rate is taken.<br>Your height will be checked at your first visit.<br>For all other visits, you must be lying down for at least 1 minute before your blood pressure, heart rate, temperature, and breathing rate is taken.
ECG	A standard 12-lead electrocardiogram (ECG) will be done (sticky patches will be placed on your chest to measure your heart's activity) at the first visit. Three ECGs will be done 5 to 10 minutes apart, 19 times during the study. You will be lying down until the final ECG is finished.
Sputum tests	You will be required to collect and provide your sputum as instructed during the study to check the number of TB bacteria growing in your body and to find out if the treatment is effective against those TB bacteria. One sample will be collected at the beginning of the study, 2 samples will be collected on your second visit, and up to 3 samples will be collected at all other visits till the end of the study.<br>On the days when three sputum specimens are collected:<br>• The first sputum will be collected at home when you wake up in the morning and before your morning meal.<br>• The second sputum will be collected at the trial site into an empty sample cup.<br>• The third sputum will be collected into a tube with liquid that helps protect parts of the TB bacteria. This sputum will be frozen, shipped, and tested in Colorado, USA to find out if the treatment is effective.<br><br>If TB bacteria grow from your sputum, those bacteria will be frozen and stored in the freezer. They may be tested in the future to see how the treatment affects the bacteria, and to see if the bacteria change over time.<br><br>If you are unable to give a sputum sample at home, this will be collected as soon as you get to the trial site. If you cannot cough up a sputum sample, the doctor may ask you to breathe in a salt water mixture to help you cough.
PK Blood tests for study medication levels (OPC-	If you are assigned to OPC-167832, Delamanid and BDQ, blood samples are taken to check your blood drug concentration levels. Each PK blood draw will be about 12.5 ml (2 ½ teaspoons).

167832, Delamanid, DM-6705, BDQ and M2)	On day 1, week 8 and week 17, you will have blood taken twice: once before you take your study medication, and again 2 to 8 hours later. This is a total of about 25ml (5 teaspoons) in one day.<br>At week 2, week 4 and week 13, blood samples will be taken once before you take your study medication. This is a total of about 12.5ml (2 ½ teaspoons).<br>There are no PK blood draws if you are assigned to Rifafour.
Blood for transcriptomics (gene testing called transcriptomics test)	Blood samples 4 ml (1 teaspoon) will be collected 11 times during the study. The sample will be stored and tested at sent to the South African MRC's Genomics Centre in Cape Town for testing during the study.<br>Genetic material, also called DNA or RNA, is usually obtained from a small blood sample. Genes are found in every cell in the human body. Our genes determine what we look like and sometimes what kind of diseases we may be susceptible to. The RNA will be removed from your blood sample and the information we learn from testing may help make new tests or treatments for TB in the future. You will be asked to sign a separate informed consent form with more details about the genetic test.
<u>Genotyping</u>	Genotyping is a test that checks if the same bacteria or a new bacteria has caused your TB while you are in the study. If TB bacteria grow from your sputum, those bacteria will be frozen and stored in the freezer. They may be tested with genotyping and compared to see if the bacteria changed over time.

Please speak to your study doctor if you are concerned about the risk of radiation exposure from the X-ray or PET/CT scan.

For this study to be successful, it is important that you cooperate fully with the study doctor and follow his or her instructions precisely. Please inform the study doctor of all the medicines that you are currently taking. Do not take any other over-the counter, prescription, herbal, traditional medicines or vitamins, without first informing the study doctor.

5. What will happen to my blood, urine and sputum samples?

During the study, your blood and urine will be tested to ensure that your body continues to work well. These are tests for chemistry and haematology as well as urine tests and will be done 10 times during the study if you are assigned to OPC-167832, Delamanid and BDQ, and 11 times if you are assigned to Rifafour. The study doctor will check the test results.

Your blood, urine and sputum samples will be sent via a courier to the following laboratory for testing:

Cytespace Africa Laboratories
125 Amkor Road
Lyttelton Manor
Centurion, 0157
South Africa

The study doctor will receive the results of the blood, urine and sputum tests.

If the laboratory cannot test your specimens for some reason, the specimens may be sent to another laboratory that is certified to perform the tests.
Your plasma (the clear, straw-coloured liquid portion of blood) from the PK blood samples will be checked at the following laboratory outside of South Africa for blood drug concentration levels:

Labcorp Bioanalytical Services3301 Kinsman Boulevard
Madison, WI 53704,
USA

Blood samples for transcriptomics will be sent to the following lab for testing:

South African MRC's Genomics Centre
Francie van Zijl Drive
Parowvallei
Cape Town
South Africa

The third sputum collected in the protective liquid will be sent to the following lab:

University of Colorado Denver
University of Colorado Denver Pulmonary Division, Research 2
12700 E. 19th Avenue
Aurora, CO 80045
Colorado
USA

Some TB bacteria from your sputum samples may be sent to the following lab for testing:

National Institute for Communicable Disease (NICD)
1 Modderfontein Road
Sandringham
Johannesburg, 2131
South Africa

Sputum specimens and material derived from the sputum specimens may be stored for up to 50 years after the study has finished for testing at Cytespace Lab. For these stored samples, we will use your study ID number instead of your name on the samples. If the laboratory is unable to test the sputum specimens or TB bacteria, the sponsor may move these specimens to another laboratory that is certified to perform the testing.

6. What are the possible risks or side-effects of being in the study?

- **<u>OPC-167832</u>**
  To date, the study medication has been studied in animals (rats and dogs) and in two clinical trials in humans.

  The potential side effects reported in the human studies conducted were headaches (pain in the head) and pruritus (itchy skin). These are the most commonly reported side effects. Arthralgia (painful joints) and ecchymosis (bruising) were also reported.

  Other possible side effects of the study medication in humans can only be predicted based on the results from studies in rats and dogs. Some of the side effects in rats and dogs when given very high doses of OPC-167832 included: changes in body chemistry tests (like cholesterol), appetite and weight changes, muscle fibre changes and changes in electrical activity of the heart. Currently there is not sufficient evidence to believe that any of the side effects listed are expected in humans.

- **<u>Delamanid</u>**
  The most common side effects include:
  Nausea, vomiting (feeling and being sick), and headache.

  Other common side effects include: abdominal pain, abdominal discomfort, dyspepsia (heartburn), gastritis (inflammation of the stomach lining), decreased appetite (not wanting to eat), asthenia (weakness or lack of energy), insomnia (difficult falling asleep) ,depression

(feeling sad), anxiety (feeling anxious), psychotic disorder, hallucination, tinnitus (ringing in the ears), blurred vision, irritation in the throat, excessive sweating, low potassium levels in the blood, dizziness (feeling dizzy), paraesthesia (a pricking or tingling sensation), hypoaesthesia (reduced feeling of touch or sensation), tremor (shaking), palpitations (feeling of fast heart beat), chest pain, and atrioventricular block 1st degree (usually slower conduction of electrical impulses in the heart) and electrocardiogram QT prolonged (indicating possible changes in your heart rhythm).

The most important safety concern is QT prolongation, a disturbance in the heart's electrical activity that can lead to serious heart rhythm disturbances and sometimes to sudden death. However, in the studies and safety data from use of the drug to date, no clinically relevant events occurred as a result of this prolongation.

- **<u>Bedaquiline (Sirturo®)</u>**
  Side effects associated with Bedaquiline (BDQ):
  - Serious heart rhythm changes (QT prolongation) on ECG which may present as change in heartbeat (a fast or irregular heartbeat) or fainting and in serious and rare cases may lead to Torsades de pointes (life threatening condition).
  - Liver problems which may present as nausea or vomiting, abdominal pain, fever, weakness, itching, being unusually tired, loss of appetite, light coloured bowel movements, dark coloured urine, yellowing of your skin or the white of your eyes (jaundice).
- Other common side effects include headache, chest pain, joint pain, feeling dizzy, feeling sick (nausea or being sick (vomiting), diarrhoea, increased liver enzymes in your blood tests, aching muscles, tender or weak muscles (not caused by exercise).

- **<u>Rifafour® tablets</u>** are a combination of four medicines (Rifampicin, Isoniazid, Ethambutol and Pyrazinamide) used to treat TB. These medications are the regular medication (standard of care) used in South Africa for the treatment of TB and have the following known side effects**:**

  Side-effects associated with rifampicin:
  - Some participants may experience a skin rash which presents 2 to 3 hours after a daily or intermittent dose i.e. facial flushing (redness of the face), itching, rash, eye irritation.
  - A 12 hour "flu" syndrome, with fever, chills, bone pain and malaise (tiredness).
  - Gastrointestinal effects include nausea (feeling sick), vomiting (being sick), anorexia (not eating), diarrhoea (loose stools) and stomach ache, which may be prevented by eating food.
  - There have been reports of pseudomembranous colitis (swelling of the intestine).
  - Hepatitis (liver disease), nausea (feeling sick), vomiting (being sick), unusual tiredness (feeling very tired)).
  - Rifampicin can cause thrombocytopenia (low platelets in the blood) and purpura (rash with purple spots on the skin)
  - Other haematological (blood) adverse effects include eosinophilia, leucopenia (low white cell count) and haemolytic anaemia (low red blood cell count).
  - Nervous system effects include headache (sore head), drowsiness (feeling tired), dizziness (feeling dizzy), ataxia (loss of control of body movements), numbness (no feeling in a body part), visual disturbances (not seeing clearly) and muscular weakness.
  - Changes in kidney function and renal failure have occurred.
  - Rifampicin may cause orange-red discoloration of urine and other body fluids, like sweat, saliva, and tears.
  - Menstrual disturbances in women have been reported (Monthly periods may change).

  Side-effects associated with isoniazid:
  - Elevated liver enzymes associated with clinical signs of hepatitis such as nausea (feeling sick), vomiting (being sick) or fatigue (feeling tired).

- Gastrointestinal effects (nausea/feeling sick), vomiting (being sick), pellagra (lack of Vitamin B3) and hypersensitivity reactions (skin eruptions (rash)
- Low red and white cell blood counts.
- Neurological effects include psychotic reactions (seeing things and feeling strange) and convulsions (having fits) peripheral neuropathy (tingling and burning of the feet), ataxia, and paraesthesiaThe risk of neurologic side effects can be significantly reduced by also giving you pyridoxine (vitamin B6).
- Optic neuritis (swelling that damages the nerve in the eye) has also been reported.

Side-effects associated with pyrazinamide:

- The most serious side-effect is hepatotoxicity (liver damage) and its frequency appears to be dose-related.
- Hyperuricaemia (high uric acid in the blood) commonly occurs, occasionally accompanied by arthralgia (painful joints) and may lead to attacks of gout.
- Photosensitivity (oversensitivity of skin to light) and skin rash have been reported less frequently. Other side-effects that have been reported are anorexia (do not want to eat), nausea (feeling sick) and vomiting (being sick), malaise (feeling tired), fever (high temperature), sideroblastic anaemia (low blood count) and dysuria (feeling uncomfortable or painful to pass urine).

Side-effects associated with ethambutol:

- Retrobulbar neuritis (swelling in the eye) with a reduction in visual acuity (difficulty to see clearly), constriction of visual field (difficulty to see clearly), central or peripheral scotoma (difficulty to see clearly), and green-red colour blindness may occur, affecting one or both eyes. Difficulty seeing seems to depend on the dose and length of treatment.
- Retinal haemorrhage (bleeding in the eye) has occurred less frequently.
- Renal clearance of urate may be reduced (passing salt or uric acid in urine may be less) and may cause acute gout.
- Hypersensitivity reactions include skin rash, pruritic (itching of the skin), leucopenia (low white cells in the blood), fever (high temperature) and joint pains.
- Gastrointestinal disturbances include metallic taste (metal taste in the mouth), nausea (feeling sick), vomiting (being sick), anorexia (not wanting to eat) and abdominal pain (stomach-ache).
- Other adverse effects: Confusion (not know who you are), disorientation (not knowing where you live), hallucinations (seeing things that are not there), headache (sore head), dizziness (feeling dizzy), malaise (feeling tired), jaundice (yellowing of the skin) or transient liver dysfunction (liver not working properly for a short while), peripheral neuritis (damage to the nerves).

- **Radiation Risk:**

  Chest X-Rays

  The risks associated with the chest X-ray are minimal due to the small amount of radiation you are exposed to in a single X-ray. Chest X-rays usually have no side effects in the diagnostic range unless you are exposed to a great number over your lifetime. There is a slightly greater risk to the reproductive cells (i.e., egg and sperm cells) of adults and to the developing foetus in the womb. There are no associated discomforts during a chest X-ray, the imaging is fast and easy, and no radiation remains in the body after an examination.

  PET/CT Scan:

  This research study involves exposure to radiation from 3 PET/CT scans. This radiation exposure is not required for your medical care and is for research purposes only. You will be asked to sign a separate informed consent form with more details about the PET / CT scan. You

may still participate in the study if you decide that you do not want to have the PET / CT scans performed.

- **Blood tests:**
  For your safety, you will have blood tests. Blood will be drawn through a small needle placed in your vein. At the area where the blood is taken, there may be mild pain, bruising and swelling. More rarely, you may faint, or the area may become infected.

- **Electrocardiogram (ECG):**
  The ECG is a procedure that requires you to lie still for a few minutes while electrodes are attached to your chest to record the activity of your heart. The ECG leads placed on your skin may cause slight discomfort during placement and removal. Some individuals are sensitive to the sticky patches used during an ECG, which may result in redness and sore skin in those areas.

- **Other risks:**
  All medications have the risk of an allergic reaction that could become life threatening. You must report all problems and worries to a member of the study staff.

During this study you will be observed for any bad or harmful effects. The study doctor will decide if it is safe for you to continue in the study. You must inform the study doctor immediately if you experience any negative effects, complications or injuries while taking the study medicine.

There may be risks with the use of the study medicine that are not currently known. You will be notified of any new significant findings that may affect your willingness to continue in the study. Only you, the study participant, can take the study medicine. If the study medicine is not effective, and the TB bacteria growing in your body does not decrease, you will probably not receive any benefit by participating.

7. Risks to women of childbearing potential

It is not known if the OPC-167832, Delamanid and BDQ can be harmful to unborn/breastfed babies. Therefore, you are not allowed to enter the study if you are pregnant**,** breastfeeding or trying to become pregnant. Women of childbearing potential will be required to undergo pregnancy tests at the first visit. If you are able to have children and you are sexually active, you must agree to use 2 different approved methods of birth control or remain abstinent (abstinence as voluntary chosen lifestyle) throughout the study and for 12 weeks after the last dose of OPC-167832, Delamanid and BDQ or RHEZ.

The following acceptable methods of contraception are:
- Bilateral tubal ligation or sterilization
- Injectable contraception, which is given every two months (eight weeks) called Nur-Isterate and Depo Provera or Petogen (DMPA) which is given every three months (12 weeks).
- Oral contraception for women (the pill) which come in a packet of 28 and you should take them once daily at the same time. There are two main types: combined oral contraceptive (COC) pills and progestogen-only contraceptive (POP) pills.
- IUD or Intrauterine Device which is a small device that is put into a women's uterus (womb) by a specially trained health worker
- Hormone patch which is a sticker with three layers. It thickens your cervical mucus and prevents sperm from entering your womb.
- Long-acting progestogen implants which are put under the skin and last for between 3 – 5 years.
- Male and female condoms. Condoms are free of charge at clinics.

No other contraception will be allowed.

Male participants must agree to use 2 different approved methods of birth control or remain abstinent throughout the participation in the study (abstinence as voluntary chosen lifestyle) and for 12 weeks after the last dose of study medication, Delamanid and BDQ or RHEZ.

Where applicable, if you are a male participant with a female partner of childbearing potential, you and/or your partner must agree to use the abovementioned acceptable contraceptive measures.

If you or your partner become pregnant during the study, you must stop the OPC_167832, Delamanid and BDQ and inform the study doctor immediately. If possible, you should remain in the study and complete the applicable study procedures. With your permission, the pregnancy will be followed up by the study team. The study doctor will advise you about you and your baby's future medical care.

Male participants must agree not to donate sperm from trial screening through 12 weeks after the last dose of OPC-167832 Delamanid and BDQ or RHEZ.

8. What are the possible benefits of being in the study?

There are no direct benefits expected from being in this study. The information gained during the study may benefit society by gaining useful information on how safe, well tolerated, and effective the study medication is when given with Delamanid and BDQ and compared to Rifafour for the future treatment of TB.

9. Alternative treatments

Instead of taking part in this study, you may choose to receive standard treatment with other medication that is available to treat your condition. The study doctor will discuss the risks and benefits of these standard treatments with you. You do not have to participate in this study to receive care and treatment for your condition and may be treated by your local clinic or doctor.

If you were assigned to OPC-167832, Delamanid and BDQ and the treatment is not working, the study doctor will change your treatment to the standard treatment for TB in South Africa. This is Rifafour. Or, if further treatment is required your doctor will discuss this with you.

- If your sputum results show that you still have TB at the end of your study treatment
- If your sputum results have been negative and then your sputum results later become positive for TB
- If, the study doctor thinks you are not getting better after examining you and/or looking at your chest X-rays

10. Physical Injury Resulting from Participation

You agree to join this study of your own accord. You should report any discomforts, problems, or research related injuries immediately to your doctor at *Telephone number* _________________. If you are injured because of being part of this study, your doctor will provide usual medical care. The research study is covered by an insurance policy taken out by Otsuka Pharmaceutical Development & Commercialization, Inc., Rockville, MD 20850, United States in the event that you suffer a bodily injury as a result of taking part in the study.

The insurer will pay for all reasonable medical costs required to treat your bodily injury, in accordance with the SA Good Clinical Practice Guidelines (2020), which are based on the Association of the British Pharmaceutical Industry Guidelines. You may request a copy of these guidelines from the study doctor.

The insurer will pay without you having to prove that the research was responsible for your bodily injury.

The insurer will **not** pay for harm if, during the study you:

- Use medicines or substances that are not allowed
- Do not follow the study doctor's instructions
- Do not tell the study doctor that you have a bad side effect from the study medicine
- Suffer an injury arising from negligence on your part or do not take reasonable care of yourself and your study medicine.

If you are harmed and the insurer pays for the necessary medical costs, usually you will be asked to accept that insurance payment as full settlement of the claim for medical costs. However, accepting this offer of insurance cover does not mean you give up your right to make a separate claim for other losses based on negligence, in a South African court.

If you belong to a private medical scheme, you should inform them that you are participating in a research study.

11. Confidentiality

If you join the study, personal information about you will be collected by the study doctor and study staff and used for the study. We will refer to this personal information as "Information." This section describes how your Information will be used and with whom it may be shared. It also describes your rights with respect to this Information.

Your Information
Information is personal information that identifies you or could be used to identify you. The study doctor and staff will collect Information from your health records to understand your medical history. As the study continues, they will also collect Information resulting from your participation.
The following are examples of Information that may be collected:

- Your name, address, contact information, birth date, gender, and identification numbers.
- Your medical history, including health conditions, treatments, and medical procedures.
- Your heart rate, blood pressure, results of study tests, and results of tests on your blood and other samples.
- Results of examinations and laboratory tests, including blood tests and medical imaging.

Uses of Your Information
Your Information will be used for the purposes of this study. This includes, for example, to determine if you are eligible for this study; to evaluate how your health changes during the course of the study; to compare changes in health during the course of the study with that of other study participants; to assess the safety and usefulness of any study drug included in the study; to learn more information about the disease(s) or health conditions(s) that are the subject of the study; to reimburse you to help cover costs associated with your study; and to provide you with treatment in the event of a study-related illness or injury.

Disclosure of Your Information
The study doctor and staff will send your Information to Otsuka Pharmaceutical Development & Commercialization, Inc *(OPDC)*, who is the sponsor of this study, and to representatives, designees and personnel working on behalf of *OPDC. OPDC* may also share your Information with its affiliates, as well as with business partners, including companies, research institutes, or universities, with whom it is working to jointly conduct medical research.

Your Information may also be shared by the study doctor and staff and by *OPDC* personnel and its designees and affiliates of *OPDC* including researchers, the Institutional Review Board or Ethics Committee overseeing this study, and regulatory authorities, including the FDA, European Medicines

Agency (EMA; the European counterpart to the FDA), and any other regulatory authorities in countries that have similar requirements. These people will check your Information, and your health record, to confirm the study is being done correctly. They may copy Information from your health record.
Your Information will also be disclosed as part of the documents reviewed by regulatory authorities for marketing approval. These regulatory authorities may require that we make certain documents and results or data relating to this study publicly available. However, your Information will be provided in combination with all other trial participants as a part of a larger data set or your Information will be redacted and made anonymous, so Information that may identify you specifically will not be publicly available. Your records will be kept as private as possible under the law.

The collection and presentation of your Information to *OPDC*, the FDA, EMA, or similar foreign regulatory agencies will be done in compliance with all applicable standards of privacy.

Your Information will be included as part of the summary results of this trial which may be disclosed in medical journals or academic conferences. The results data collected may also be shared with other researchers who want to use the data to conduct additional analysis. In the event that results of this study are disclosed as described, your Information will be made anonymous, and will be provided in combination with all other trial participants as a part of a larger data set.

A description of this clinical trial will be available on http://www.ClinicalTrials.gov, as required by U.S. Law. This website will not include information that can identify you. At most, the website(s) will include a summary of the results. You can search this website(s) at any time. In addition, a description of this clinical trial will be available on https://sanctr.samrc.ac.za.

Your Information may also be sent to, stored at, or used outside of your country. Although confidentiality rules in other countries may be less strict than in your country, even if your Information is transferred to a different country, it will always be handled in accordance with applicable law. OPDC, the sponsor of this trial is the data controller. The data controller, alone or jointly with others, determines the purposes and means of the processing of your Information.

Data Protection
Your records will be kept as private as possible under law. Your Information will be stored in limited-access databases. The study doctor and staff will have access to these databases. Each of these individuals will be obligated to protect the confidentiality of your Information and to use and disclose it only as described in this document.

The study doctor and staff will replace personal information that directly identifies you (e.g., your name and contact information) with a code before reporting Information to *OPDC*. *OPDC*'s employees and representatives are required to protect your personal data, not to attempt to re-identify the data, and to use your Information only for the purposes described above. In addition, *OPDC* has implemented security measures to prevent unauthorized individuals from accessing your Information.

While these measures reduce the risk of your Information being misused or accessed by unauthorized individuals in a form that identifies you or that can be easily re-identified, such risks cannot be entirely eliminated. In the event your Information were to be accessed by unauthorized individuals, medical information about you could be of interest to employers, insurers, or law enforcement, and could be used to make decisions which may be averse to you. Although we believe the risks of re-identification and adverse decisions are low, we cannot describe all possible harms to you at this point. You should know that many, but not all, possible adverse uses of your re-identified or improperly disclosed medical information are prohibited by medical ethics, law, or regulation.

Data Retention
Your Information will be stored for at least 10 years, in accordance with applicable law. At the end of that time, your Information will be destroyed.

Your Data Rights

You have the right to withdraw your permission for use of your Information for this research study. If you wish to withdraw your permission, you must notify the study doctor or study staff. Once permission is withdrawn, you cannot continue to take part in the study.

If you withdraw your permission, no further information will be collected about you. However, the Information collected before you withdraw will still be used. Information that has already been used or has been shared with others cannot be taken back. Information that is already part of larger datasets or have been or are being shared for further research cannot be removed.

May I get access to my Information?

You have the right to see and get a copy of your Information. If you believe any of your Information is inaccurate or incomplete, you have the right to request corrections. Please note that your access to your Information may be suspended during your participation in the study. Therefore, if you desire immediate access to your Information, you may not be able to continue participating in the study. In order to request a copy of your Information or to exercise your rights of access or correction, you may contact *Name of Study Doctor* ____________________ at *Telephone number* __________________. Please indicate in what format you wish to receive a copy of your Information (e. g, paper copies, electronic format) or if you wish to request the direct transfer of your Information to a third party.

There may be legal and regulatory exceptions to the amount of information that responding parties may disclose. In addition, you have the right to request information from *OPDC* on how your Information is being used and with whom Information has been shared. You can also request the deletion of any part of your Information that is no longer needed or the restriction of its use. Please be aware that because *OPDC* only maintains coded study data, *OPDC* may not be able to fully respond to your request, but *OPDC* will try to be as responsive as possible. In order to make such a request, you may contact *Name of Study Doctor* ___________________ at *Telephone number* at the study site and request that the study site forward your request to *OPDC*.

If you have questions, concerns, or complaints as to how *OPDC* is using your Information, you can contact *OPDC*'s Data Protection Officer at DPO@otsuka-us.com. Finally, the South Africa Data Protection Authority is responsible for making sure that privacy law is followed in South Africa.

By signing this form, you are confirming that you consent to the collection, use, and disclosure of your Information as described in this document. You are also confirming that you understand that there are potential risks to your privacy by authorizing the collection, use, and disclosure of your Information for this study.

All personnel accessing your records are required to respect your confidentiality at all times. Representatives from government agencies such as the South African Health Products Regulatory Authority, the National Health Research Ethics Council (NHREC) and the <*Name of Ethics Committee*>, the Sponsor and or the Sponsor's authorized representatives may need access to your original medical records and study records to confirm that the study data collected about you is correct and relates to you.

We will keep all information from your medical records as private as the law allows. The National Health Act, Act No.61, of 2003 in South Africa ("National Health Act") protects the confidentiality of citizens' medical records. However, you should know that the National Health Act allows release of some Information from your medical records without your permission, for example, if it is required by a court order or by law. Part of this National Health Act also requires that certain infectious diseases be reported to the health authorities. One of the diseases that must be reported is TB. The study team will, therefore, make sure that your TB clinic is aware of your case of TB.

Confidentiality regarding your HIV test results: No blood samples for the HIV test will be stored for any reason and your HIV testing information and test results cannot be released to anyone without your

written consent. Your study doctor will record your HIV test result information in your study health records for use by OPDC personnel and its designees. General consent to health care and information release does not cover HIV-related information. If you are found to be HIV infected, you are personally not required to tell anyone about this diagnosis. However, it is very important to notify your sexual partners and those who might have been exposed to your blood.

12. Payment, expenses and costs

You will not receive payment for participating in this study.

You will be reimbursed for your transportation to and from the trial site, loss of income for the days you have to visit the study clinic, inconvenience, and any expenses you may have during the day of your visit.

You will receive R 440.00 per visit to reimburse you for such expenses. If your study-related costs exceed this amount and you have proof of such expenses, please discuss them with your study doctor.

13. Termination of participation

Your participation in the study may be stopped for the following reasons:

- If you don't follow the study doctor's instructions.
- If you do not take the study medicine as prescribed.
- If the study doctor decides that it is in your best interests.
- If there aren't enough participants in the study, or the study has acquired the required number of participants.
- If the sponsor stops the study or closes the study site for any reason.
- If the sponsor stops the dose group you are in, for any reason.
- If you fall pregnant.

You will be asked to complete an early termination visit if you are still on study treatment. During this visit the following procedures will be done. Chest Xray, vital signs, physical examination, ECG and bloods, a urine and sputum samples will be taken.

In this study, it is very important to follow your progress and health even if you do not want to continue taking the study medicine. If you want to stop taking the study medicine, your study doctor will ask you the following questions:

- Would you be willing to continue to participate in the study tests and procedures as described in this Patient Information Sheet that would normally be done during the remaining planned course of the study duration?
- May the study staff continue to use your samples/data for future analysis as described in the Use of Your Information (section 11), Use of Study Samples (section 5) and Confidentiality (section 11) of this Patient Information Sheet?
- May the study staff continue to have access to your information as it relates to your disease and to have the study staff access your medical records as related to the study aim?
- May the study staff contact you to follow up on your condition until the end of the study duration?
- May the study staff contact a third party(ies) who have been designated in source records as being designated to discuss your medical condition, by telephone, mail or email (e.g. family, spouse, partner, legal representative, friend, neighbor, physician)?
- May the study staff obtain your medical information from alternative sources (e.g. hospital/clinic medical records, referring doctor's notes, public records, dialysis, transplantation or vital registries, social media sources)?

You will not be considered as having fully withdrawn consent from the study unless you answer "No" to all of the questions above.

Your continued participation in tests, the collection of follow up and health status information, the use of your sample/s data and the use and disclosure of your health information is voluntary. You have the right to withdraw consent to any or all of the questions above at any time by notifying the doctor in writing or verbally in person, via phone and the doctor will record your decision in writing.

14. Study results

You will be able to obtain information about your study results and the outcome of the study by contacting *Name of Study Doctor* ___________________. Tel: ____________________

In addition, you may check the South African National Clinical Trials Registry website https://sanctr.samrc.ac.za where participants can obtain information on the trial.

15. Contacts for answers relating to the research, and your rights as a research participant in the event of trial-related injuries or side-effects

Study-related injury (your rights as a research participant)

< Name of Ethics Committee>
<Address and telephone number, fax number and email address for the Ethics committee>

The Chair
National Health Research Ethics Council
Phone: 012 395 8119
Fax: 012 395 8467
e-mail: Tshilidzi.Muthivhi@health.gov.za

If you have questions about this trial, you should first discuss them with your doctor or the Ethics Committee (contact details as provided on this form). After you have consulted your doctor or the Ethics Committee and if they have not provided you with answers to your satisfaction, you should write to the South African Health Products Regulatory Authority (SAHPRA) at:

The Chief Executive Officer
South African Health Products Regulatory Authority
Loftus Park
Building A
402 Kirkness Street
Arcadia, Pretoria
0083
E-mail: Boitumelo.Semete@sahpra.org.za
Tel: 012 501 0413

16. Consent statement

By signing below, I agree that: **Initial Blocks**

• I have read or had read to me the information sheet and consent form, version 5.0_03Feb2023 for this study.	
• I understand that this trial is investigational and what investigational means.	
• The purpose, treatment and procedures of this trial have been explained to me and I understand them.	
• I understand my responsibilities as a trial participant.	
• I understand that participation in the trial is voluntary and that I can refuse to participate or withdraw at any time, without it affecting my ongoing care.	
• I have been informed of the possible risks, harm and inconvenience of participating.	
• **For women:** I am not pregnant, breastfeeding or trying to fall pregnant and will use acceptable birth control during the trial. **For male participant with a female partner of childbearing potential:** My partner and I agree to use the acceptable contraceptive measures.	
• I have been informed of the expected benefits of the trial.	
• I have been informed of the alternative treatment, including its potential risks and benefits that may be available to me if I do not participate in this trial.	
• I have been informed of the compensation and treatment that would be available to me in the event of a trial-related injury.	
• I have been informed of any reimbursement I may receive, as well as any anticipated expenses that I may incur while participating in the trial.	
• I have had sufficient time to ask questions and they were answered to my satisfaction.	
• I have been given time to discuss the trial with others and to decide whether or not to take part.	
• I am aware that the results of the trial, including personal details about me and my health information may be reasonably disclosed to the sponsor, regulatory authorities and research ethics committees, if required by law.	
• I agree for my blood and sputum to be sent to the material derived from my sputum samples to be transferred to a secure central laboratory outside South Africa.	
• I will receive a signed and dated copy of this informed consent form.	
• I agree to participate in this trial.	

____
Printed name of participant

____
Signature of participant

____
Date (personally completed by participant)

____

Printed name of person conducting consent (if other than the Investigator)

____

Signature of person conducting consent (if other than the Investigator)

____

Date

____

Printed name of Investigator

____

Signature of Investigator

____

Date

I hereby verify that verbal consent was obtained from the above participant. The participant has been informed about the risks and the benefits of the research, understands such risks and benefits and is able to give consent to participate, without coercion, undue influence or inappropriate incentive.

____

Printed name of witness (*Witness signature is required in instances where the participant is illiterate. Verbal consent must be obtained from the participant, in the presence of an independent witness who is present during the entire informed consent discussion. The witness's name, signature and date must be completed by the witness at the same time when consent is obtained by the participant and the document is signed and dated by the study doctor/delegate. A competent witness for research purposes is a person who is 18 years or older, and of sound mind and who is not involved with the trial in any way)*

____

Signature of witness

____

Date
